# Analysis of changes to lncRNAs and their target mRNAs in murine jejunum after radiation treatment

**DOI:** 10.1111/jcmm.13940

**Published:** 2018-10-16

**Authors:** Qianying Lu, Wei Gong, Jinhan Wang, Kaihua Ji, Xiaohui Sun, Chang Xu, Liqing Du, Yan Wang, Qiang Liu

**Affiliations:** ^1^ Institute of Radiation Medicine Chinese Academy of Medical Sciences & Peking Union Medical College Tianjin Key Laboratory of Molecular Nuclear Medicine Tianjin China

**Keywords:** alternation, intestinal injury, lncRNAs, mRNAs, radiation

## Abstract

LncRNAs have been reported to play an important role in various diseases. However, their role in the radiation‐induced intestinal injury is unknown. The goal of the present study was to analyse the potential mechanistic role of lncRNAs in the radiation‐induced intestinal injury. Mice were divided into two groups: Control (non‐irradiated) and irradiated. Irradiated mice were administered 14 Gy of abdominal irradiation (ABI) and were assessed 3.5 days after irradiation. Changes to the jejuna of ABI mice were analysed using RNA‐Seq for alterations to both lncRNA and mRNA. These results were validated using qRT‐PCR. LncRNAs targets were predicted based on analysis of lncRNAs‐miRNAs‐mRNAs interaction. 29 007 lncRNAs and 17 142 mRNAs were detected in the two groups. At 3.5 days post‐irradiation, 91 lncRNAs and 57 lncRNAs were significantly up‐ and downregulated respectively. Similarly, 752 mRNAs and 400 mRNAs were significantly up‐ and downregulated respectively. qRT‐PCR was used to verify the altered expression of four lncRNAs (ENSMUST00000173070, AK157361, AK083183, AK038898) and four mRNAs (Mboat1, Nek10, Ccl24, Cyp2c55). Gene ontology and KEGG pathway analyses indicated the predicted genes were mainly involved in the VEGF signalling pathway. This study reveals that the expression of lncRNAs was altered in the jejuna of mice post‐irradiation. Moreover, it provides a resource for the study of lncRNAs in the radiation‐induced intestinal injury.

## 1 INTRODUCTION

The human genome contains relatively few protein‐coding sequences, while the majority is transcribed to produce non‐coding RNAs (ncRNAs). Long, non‐coding RNAs (lncRNAs) form a large class of ncRNAs that are more than 200 nt in length. Based on the relative chromosomal position of the gene, a lncRNA can be placed into one or more of five broad categories: (a) sense, (b) antisense, (c) bidirectional, (d) intronic or (e) intergenic.^1^

Although they are non‐coding, lncRNAs are able to regulate gene expression and do so through multiple mechanisms.^2^ These primarily include the following: (a) Chromatin modification, which includes lncRNAs HOTAIR,^3^ Xist^4^ and ANRIL,^5^ (b) transcriptional regulation, including Evf2^6^ and (c) post‐transcriptional regulation, including Zeb2.^7^ Moreover, lncRNAs possess a variety of biological functions, such as epigenetic regulation of gene expression^4^ as well as the production of competing endogenous RNA. Some lncRNAs also serve as host genes for small, non‐coding RNAs that are deregulated in cancer.^8^ Other disease‐relevant roles (eg, aetiology and development) have been reported for lncRNAs, such as in infectious diseases,^9^ ophthalmological^10^ as well as neurodegenerative diseases,^11^ and various cancers.^8^

More than half of the cancer patients are currently treated with radiotherapy, but this approach is inexact and often damages surrounding healthy tissues. In particular, the epithelium of mammalian intestinal mucosa undergoes rapid and constant renewal throughout the life of an organism. Additionally, it acts as a physical barrier between the luminal microbiota and the rest of the body.^12^ As its rapid renewal and does not have much natural protection, the intestinal epithelium is sensitive to ionising radiation. As such, the intestine is one of the most sensitive organs to radiation toxicity.^13^ Radiotherapy of abdominal and pelvic tumours results in high radiation toxicity that can lead to the radiation‐induced intestinal injury.

Radiation‐induced tissue injury is a complex, pathophysiological process involving multiple and wide‐ranging mechanisms, which are dependent on the radiation dose and time course. These mechanisms include DNA repair, cell death, inflammation, endothelial activation, angiogenesis and matrix remodelling.^14^, ^15^ Symptomatically, the radiation‐induced intestinal injury mainly manifests as diarrhoea, dehydration, sepsis and intestinal bleeding, with eventual mortality within 10‐15 days post‐exposure.^16^ Due to its severity, there is a tremendous need for therapeutic measures that can prevent or treat the radiation‐induced intestinal injury.

It has been reported that some lncRNAs are involved in regulating the intestinal epithelial barrier. For instance, Su et al^17^ found that miR‐874 suppressed AQP3 expression, resulting in AQP3 down‐regulation. This impaired intestinal barrier integrity and may have led to intestinal barrier dysfunction via the opening of the tight junction complex.^18^ Interestingly, H19, a maternally expressed imprinted lncRNA,^19^ may function as a competing endogenous RNA (ceRNA). This would allow for finer regulation of AQP3 expression by competing for miR‐874 and may also improve intestinal barrier dysfunction. Geng et al^20^ found that under normal conditions, H19 is transcriptionally silent in the small intestine of adult mice. However, it becomes strongly activated after LPS treatment. H19 expression is induced by IL‐22 in intestinal epithelial cells through PKA‐ and STATE3‐associated signalling mechanisms and plays an important role in sustaining the renewal of epithelial cells during inflammation, its expression also enhances regeneration of the intestinal epithelium in colitis. Chen et al^21^ devised an intestinal epithelial barrier model and used dextran sodium sulphate (DSS) to induce injury. In this model, Myc‐associated zinc finger protein (MAZ) and tight junction proteins zonula occludens 1 (ZO‐1) were involved in the functioning of the intestinal epithelial barrier. However, miR‐34c targeted MAZ to inhibit its expression, while a lncRNA PlncRNA1 protected the intestinal epithelial barrier by sponging miR‐34c. Past work has also shown that fasting for 48 hours inhibits small intestinal mucosal growth in mice. Importantly, Xiao et al^12^ compared microarray profiles between fasted and control groups and found the lncRNA uc.173 differentially expressed between the two groups. Thus, LncRNA uc.173 can enhance intestinal mucosal growth by specifically inhibiting miRNA 195 expression.

Past work has also found lncRNAs play a role in DNA damage induced by ionising radiation. For instance, Michelini et al^22^ found that following DNA damage, RNA polymerase II (RNAPII) was bound to the MRE11‐RAD50‐NBS1 complex and recruited to double‐strand DNA breaks. There, it engaged in damage‐induced synthesis of long non‐coding RNAs (dilncRNAs) directed towards both DNA ends. DilncRNAs act as DNA damage response RNA (DDRNA) precursors and also recruit DDRNAs through RNA‐RNA pairing. Together, dilncRNAs and DDRNAs help focus DNA damage response (DDR) formation and its association with 53BP1. Accordingly, RNAPII inhibition prevents DDRNA recruitment, DDR activation and DNA repair. Betts et al^23^ found that a distal transcriptional enhancer within the 11q13 breast cancer risk region (PRE1) interacted with two oestrogen‐regulated lncRNAs, CUPID1 and CUPID2 promoter. PRE1 has also previously been shown to regulate the expression of CCND1.^24^ Moreover, Betts also showed that CUPID1 and CUPID2 regulated the decision to engage either the non‐homologous end joining or homologous recombination (HR) pathway.

Past work has found many lncRNAs that are involved in intestinal epithelial barrier and DNA damage induced by ionising radiation; however, there remain limited studies examining the role of lncRNAs in the radiation‐induced intestinal injury. Currently, there is no accepted approach to either prevent or treat the radiation‐induced intestinal injury. Given this, we sought to determine which lncRNAs were involved in the radiation‐induced intestinal injury and provide a better understanding of this type of injury to guide better clinical treatment of the radiation‐induced intestinal injury. In this study, we sequenced both the lncRNAs and mRNAs in murine jejuna, both at baseline and 3.5 days post‐irradiation. We selected differently expressed lncRNAs and mRNAs, as lncRNAs can act as an endogenous “sponge” that regulates the target gene of miRNAs through competition with miRNAs, we predicted lncRNAs‐targeted miRNAs and miRNAs‐targeted mRNAs, and compared these predictions with our mRNAs sequencing results. Finally, we performed GO and KEGG signalling pathway analysis and illustrated lncRNA‐miRNA‐mRNA network; this was performed in order to find out which specific lncRNAs might be involved in the radiation‐induced intestinal injury.

## 2 MATERIALS AND METHODS

### 2.1 Mouse model of the radiation‐induced intestinal injury

Male C57BL/6 mice, aged 6‐8 weeks and weighing 23‐24 g, were purchased from Huafukang Biotechnology Co. Ltd (Beijing, China). All mice were housed in a temperature‐controlled, pathogen‐free environment with a 12‐hour light/dark cycle and allowed ad libitum access to water and standard chow. Mice had been divided into two groups: Control (non‐irradiated) and irradiated. The control group included three mice that had not been irradiated, while the irradiated group contained three mice that received 14 Gy ABI. Abdominal irradiation (ABI) was performed on mice using a Cs137 γ‐ray irradiator (Atomic Energy of Canada, Chalk River, ON, Canada). Lead shielding was used to protect other body parts from irradiation. Mice were exposed to 14 Gy at 1 Gy/min at room temperature. On day 3.5 post‐irradiation, mice were killed and the jejuna were frozen in −80°C freezer. All experimental procedures and protocols were conducted according to the guidelines of our institutional animal care and use committee.

### 2.2 Jejuna samples and RNA isolation

RNA was isolated from the jejuna of control and irradiated mice 3.5 days post‐irradiation. RNA was isolated using TRizol (Invitrogen, Carlsbad, CA, USA) according to the manufacturer's instructions. RNA concentration from all samples was determined by assessing the OD 260/280 using a NanoDrop ND‐2000 instrument (ThermoFisher Scientific, Waltham, MA, USA). RNA integrity was assessed using denaturing agarose gel electrophoresis.

### 2.3 High‐throughput sequencing

High‐throughput, whole transcriptome sequencing and subsequent bioinformatics analysis were all performed with Cloud‐Seq Biotech (Shanghai, China). The following steps were used: Total RNA was used and rRNAs were removed using Ribo‐Zero rRNA Removal Kits (Illumina, San Diego, CA, USA) following the manufacturer's instructions. RNA libraries were constructed using rRNA‐depleted RNAs with TruSeq Stranded Total RNA Library Prep Kit (Illumina) according to the manufacturer's instructions. Libraries were quantified and quality‐controlled using the BioAnalyzer 2100 system (Agilent Technologies, Santa Clara, CA, USA). Then, 10 pM libraries were denatured to single‐stranded DNA molecules, captured on Illumina flow cells, amplified in situ as clusters and sequenced for 150 cycles using an Illumina HiSeq Sequencer according to the manufacturer's instructions.

### 2.4 lncRNAs sequencing analysis

After image and base recognition, the original reads were harvested from an Illumina HiSeq sequencer. 3′ adaptor‐trimming and low‐quality removal was performed with cutadapt software,^25^ after which the resulting high‐quality clean reads were used for lncRNA analysis. Clean reads were aligned to the mouse reference genome (UCSC MM10) using hisat2 software.^26^

### 2.5 lncRNAs identification and their differential expression

Cuffdiff software^27^ was used to calculate differentially expressed lncRNAs. lncRNAs that exhibited fold changes ≥2.0 with *P* < 0.05 and FPKM value ≥0.1 in the least in one sample from a group were classified as having a significant and differentially expressed lncRNA.

### 2.6 Experimental validation of lncRNAs

Quantitative real‐time polymerase chain reaction (qRT‐PCR) was used to validate lncRNA expression. Two up‐regulated, two down‐regulated lncRNAs and mRNAs were selected for validation. The housekeeping gene GAPDH was used as a reference for normalisation. All primers used are presented in Table 1. Total RNA was reverse transcribed to cDNA using PrimeScript RT Reagent Kit (Perfect Real Time; TaKaRa, Osaka, Japan) according to the manufacturer's instructions. Resulting cDNA was then subjected to qRT‐PCR analysis on a BIO‐RAD CFX Connect Real‐Time PCR System with EVA Green qPCR Mix (Abm, Vancouver, BC, Canada). The assay was performed with three independent samples, all of which were assessed in triplicate. The relative expression ratio of lncRNAs was determined using the 2^−ΔΔCt^ method.

**Table 1:** Primers used to validate lncRNA and mRNA expression

Gene name	Primer type	Primer sequence	Product length
ENSMUST00000173070	Forward primer	GCCGTACCCAGTAGCACAAT	71
	Reverse primer	AGCAAAGCCCTTTCTTCAGGTG	
AK157361	Forward primer	TCTCAAAGCTGTTTGTGTTCTCC	84
	Reverse primer	CAAATTGTAAAGCTGAGGCTAGTCT	
AK083183	Forward primer	AGTAGTAACGATGAACTGCTGGAA	108
	Reverse primer	GGAATCTTGCTTTGACTTCACTACA	
AK038898	Forward primer	GGGAGGATTCTGTTGCGGTT	109
	Reverse primer	TAAAGGCTTCAACGGTGGCT	
Mboat1	Forward primer	AGCCTCTCTTACCGTACCACC	157
	Reverse primer	GGCTGGCTTTACCAGGATGTA	
Nek10	Forward primer	GACAGTGCCCAAAATAACATGAC	237
	Reverse primer	GGATGGAGCACGATTAACCCA	
Ccl24	Forward primer	TCTTGCTGCACGTCCTTTATT	179
	Reverse primer	GCATCCAGTTTTTGTATGTGCC	
Cyp2c55	Forward primer	TACATTTTGGGGCGAGTGAAAG	120
	Reverse primer	AAACTCCGAATGGGGATTGTG	
Gapdh	Forward primer	AGGTCGGTGTGAACGGATTTG	95
	Reverse primer	GGGGTCGTTGATGGCAACA	

### 2.7 GO and KEGG pathway analyses for selected lncRNAs

GO and KEGG analyses were performed for the differentially expressed lncRNA‐associated genes. GO analysis included three parts: Molecular function (MF), biological process (BP) and cellular component (CC). The top 10 enriched GO terms among the two groups were presented for differential expression. KEGG pathway analysis is a process that maps molecular data sets in genomics, transcriptomics, proteomics and metabolomics onto the KEGG pathway map. This mapping allows for the interpretation of biological function of these molecules. The KEGG pathway analysis was performed with differentially expressed lncRNAs and targeted mRNAs; the analysis speculated as to the pathways involved in these lncRNAs and targeted mRNAs. *P*‐value <0.05 was used as our threshold for statistically significant enrichment.

### 2.8 lncRNA‐miRNA‐mRNA network analysis

To identify the interactions between mRNAs and lncRNAs, we constructed lncRNA‐miRNA‐mRNA networks. A prediction of the different lncRNA‐miRNA interactions was made by the popular miRNA target gene prediction software, miRNA binding sites and target mRNA prediction were performed with proprietary software based on TargetScan and miRanda. Network maps for our lncRNA‐miRNA‐mRNA analyses were illustrated using cytoscape software and were based on the lncRNA, and prediction data of miRNA and mRNA.

## 3 RESULTS

### 3.1 Differentially expressed lncRNAs and mRNAs post‐irradiation

**Figure 1:**
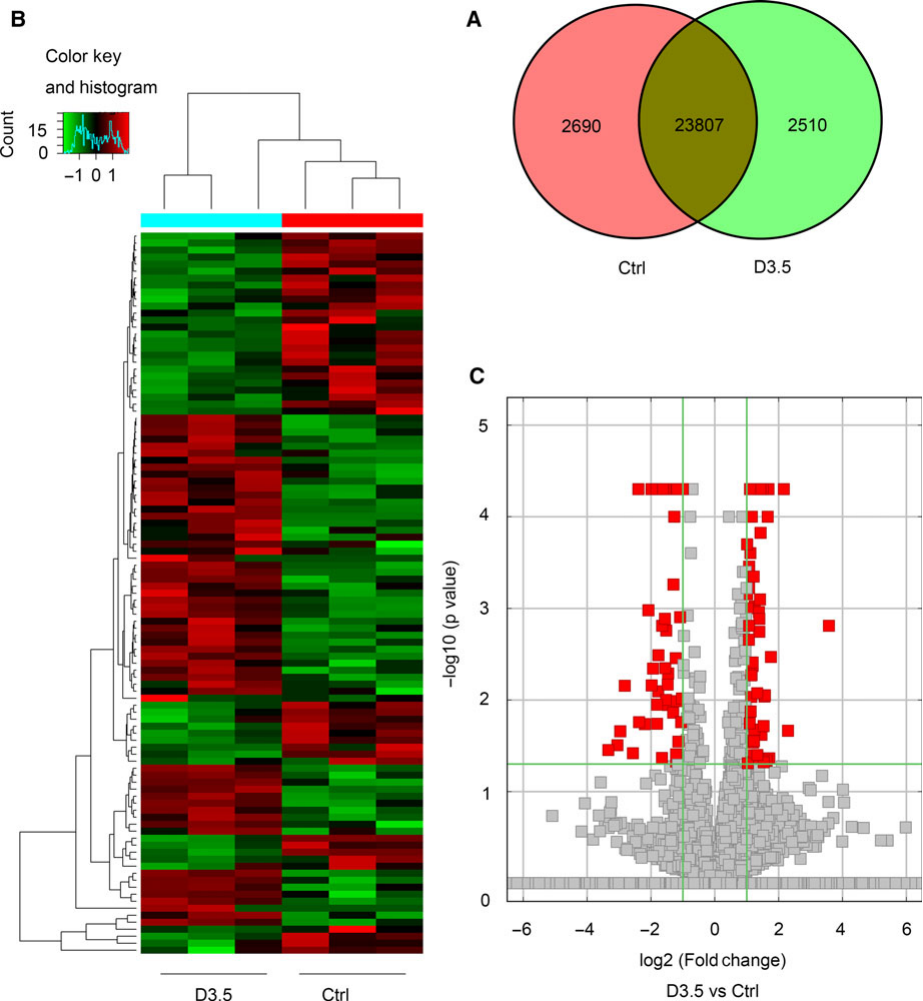
The expression profiling changes of lncRNAs in control group and irradiated group. A, Venn diagram showing the number of overlapping lncRNAs in the irradiated and control groups. B, Heat map of lncRNAs showing hierarchical clustering of altered lncRNAs in irradiated mice when compared with controls. *Red represents up‐regulation and green represent down‐regulation*. C, Volcano Plot indicating up‐ and downregulated lncRNAs in irradiated mice when compared with controls

A total of 29 007 lncRNAs and 17 142 mRNAs were detected in the control group and 3.5 days post‐irradiation. For lncRNAs, 2690 were only detected in the control group, 2510 were only detected 3.5 days post‐irradiation and 23 807 were detected in both groups (Figure 1A). Of the total 29 007 lncRNAs, 91 were significantly up‐regulated and 58 were significantly down‐regulated 3.5 days post‐irradiation when compared with controls (*P* < 0.05, fold change ≥2) (Figures 1B, C and 3A).

**Figure 2:**
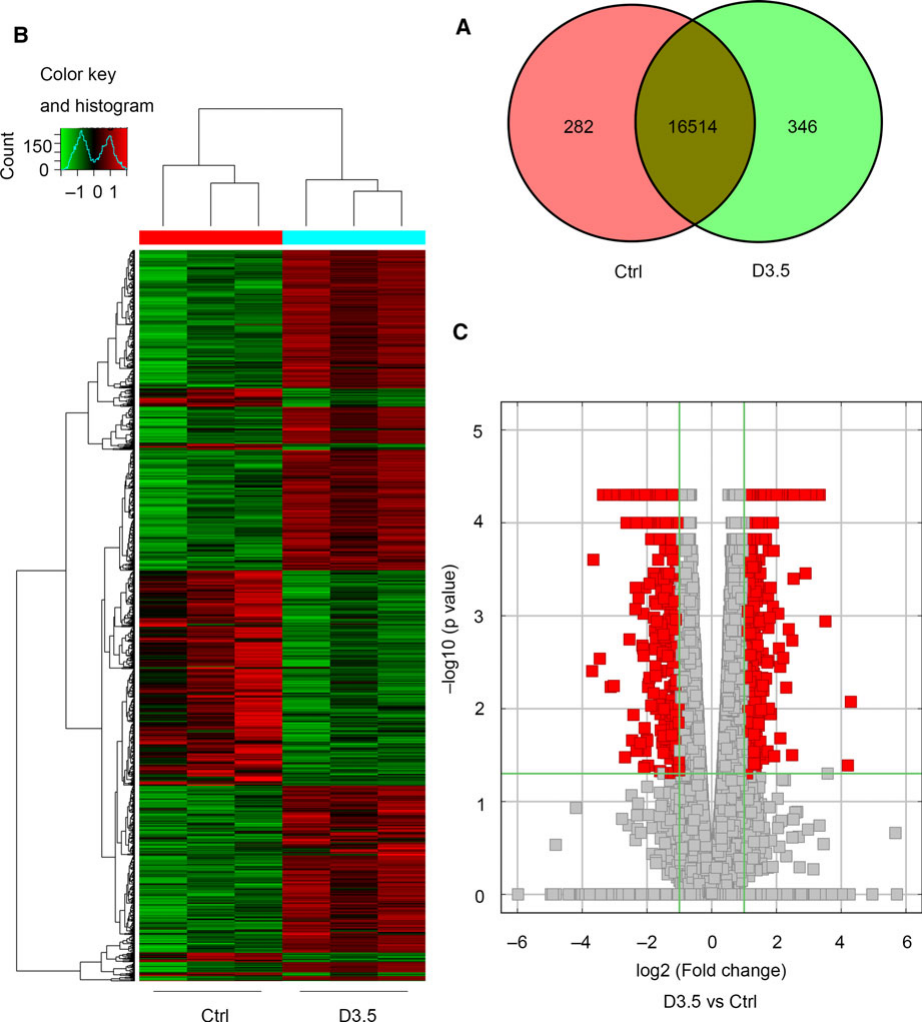
The expression profiling changes of mRNAs in control group and irradiated group. A, Venn diagram showing the number of overlapping mRNAs in irradiated and control groups. B, Heat map of mRNAs showing hierarchical clustering of altered mRNAs in irradiated mice when compared with controls. *Red represents up‐regulation and green represents down‐regulation*. C, Volcano Plot indicating up‐ and downregulated mRNAs in irradiated mice when compared with controls

**Figure 3:**
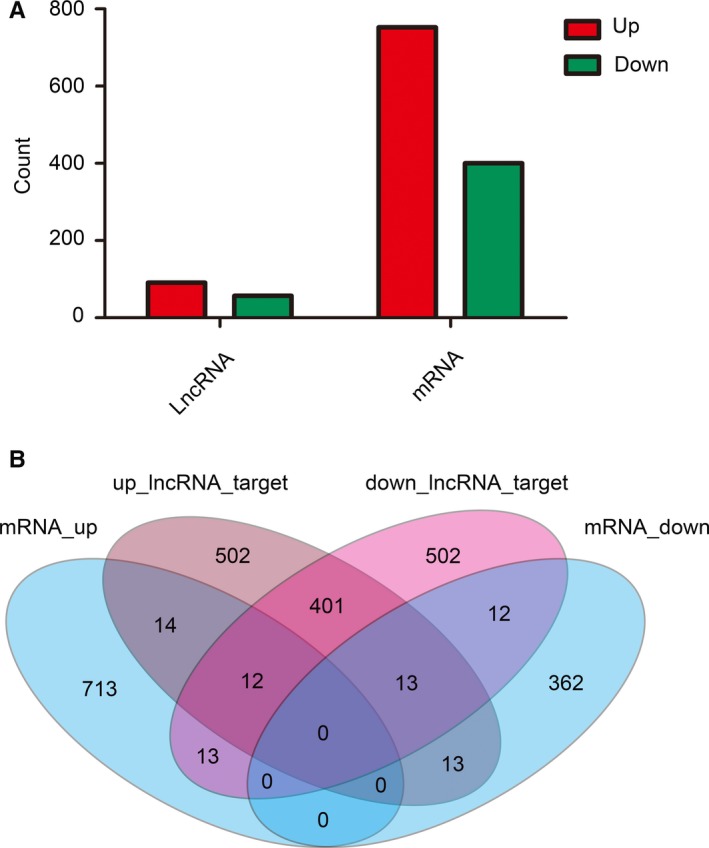
Number of relative lncRNAs and mRNAs in irradiated mice when compared with controls. A, Histogram showing the number of up‐ and downregulated lncRNAs and mRNAs in irradiated mice. B, Venn diagram showing the overlapping number of targeted mRNAs in up‐regulated lncRNAs, targeted mRNA in down‐regulated lncRNAs, up‐regulated mRNAs, and down‐regulated mRNAs

For mRNAs, 282 were only detected in the control group, 346 were only detected 3.5 days post‐irradiation and 16 514 were detected in both groups (Figure 2A). Of the 17 142 total mRNAs, 752 were significantly up‐regulated and 400 were significantly down‐regulated 3.5 days post‐irradiation when compared with controls (*P* < 0.05, fold change ≥2) (Figures 2B, C and 3A).

Using the differently expressed lncRNA data (top 20 for each of up‐regulated and down‐regulated lncRNAs are listed in Table 2), we next predicted their respective sponge miRNA and target mRNA. We also compared these predictions with our mRNA sequencing results. As shown in Table 3 and Figure 3B, 26 target mRNAs of up‐regulated lncRNAs were up‐regulated, while 25 target mRNAs of down‐regulated lncRNAs were down‐regulated. These were then used for our GO, KEGG pathway and lncRNA‐miRNA‐mRNA network analyses.

**Table 2:** Top 20 significantly up‐ and downregulated lncRNAs

Transcript ID	Regulation	Log2 FC	*P*‐value	Chr	Strand	Start	End
AK029621	Up		0.0001	9	+	108991901	109010572
ENSMUST00000182520	Up	649.341	0.04345	17			
TCONS_00013586	Up	60.8039	0.0395	17			
AK157361_1	Up	3.57644	0.00155	1			
ENSMUST00000173070	Up	2.2921	0.0217	17			
AK152734	Up	2.16347	0.00005	19			
AK013908	Up	1.7585	0.0034	13	+	108044473	108049146
NR_045710	Up	1.70723	0.0434	3			
TCONS_00025210	Up	1.68216	0.00005	5	−	23700648	23712667
TCONS_00006870	Up	1.65815	0.0001	12			
TCONS_00013542	Up	1.62523	0.0458	17	+	33909413	33911423
uc007jjl.1	Up	1.57118	0.0089	11			
ENSMUST00000183030	Up	1.55833	0.0092	9			
AK037312	Up	1.53371	0.0479	5			
AK035001	Up	1.52899	0.0193	10			
TCONS_00025212	Up	1.50848	0.00005	5	−	23700648	23712667
NR_045042	Up	1.46381	0.00005	5			
AK087220	Up	1.46334	0.0439	4	−	46379863	46389437
ENSMUST00000172531	Up	1.46145	0.02405	17			
AK085607	Up	1.43428	0.00015	9			
TCONS_00010866	Down	−7.37191	0.0053	15			
uc029sug.1	Down	−3.33117	0.03525	15			
uc009bpt.1	Down	−3.04963	0.03125	6	+	41059591	41547104
AK038898	Down	−2.95816	0.022	9			
uc009bpe.2	Down	−2.81888	0.007	6	+	41227695	41538592
TCONS_00002419	Down	−2.56474	0.03815	10			
AK083183	Down	−2.39063	0.00005	9			
2_00008059	Down	−2.36925	0.0175	13	+	44730837	44903258
TCONS_00025153	Down	−2.18865	0.01835	5			
ENSMUST00000151051	Down	−2.07808	0.00105	2			
AK156559	Down	−1.98272	0.00005	10			
uc029qtg.1	Down	−1.97477	0.00695	1	+	131797394	131818115
AK083360	Down	−1.93436	0.00455	12			
AK138493	Down	−1.87959	0.00005	2			
ENSMUST00000143673	Down	−1.82223	0.00005	11			
AK087733	Down	−1.80859	0.0182	18			
2_00001168	Down	−1.80778	0.01125	1	+	43934006	44002971
TCONS_00025152	Down	−1.76965	0.00805	5			
AK034241	Down	−1.76488	0.00325	14			
uc007zhs.2	Down	−1.65472	0.043	16	+	44765735	44794977

**Table 3:** mRNAs used for the GO and KEGG signalling pathway analyses

Gene	Regulation	Log2 FC	*P*‐value	Locus	Strand
Gm4724	Up	6.81095	0.00545	chr2:175372027‐175435807	−
Trmt10a	Up	1.41578	0.0004	chr3:138143447‐138159821	+
Polr1e	Up	1.22418	0.0023	chr4:45018582‐45084604	+
Ddah1	Up	3.51136	0.00115	chr3:144947560‐145894277	+
Ankle1	Up	1.13181	0.0008	chr8:71406009‐71409904	+
Lrp8	Up	1.38455	0.00175	chr4:107802258‐107876840	+
Ereg	Up	1.63764	0.00005	chr5:91051870‐91093649	+
Pus7	Up	1.24149	0.00015	chr5:23740647‐23783711	−
Exo1	Up	1.42521	0.00005	chr1:175880777‐175911396	+
Kif24	Up	1.35257	0.00055	chr4:41390744‐41464887	−
Celsr2	Up	1.74194	0.0006	chr3:108390850‐108415552	−
Trim6	Up	1.28095	0.00015	chr7:104218792‐104235152	+
Adamts15	Up	1.03113	0.00005	chr9:30899154‐30922452	−
Pkp1	Up	1.38649	0.00005	chr1:135871394‐135919207	−
Prrg4	Up	1.16766	0.0065	chr2:104830740‐104849876	−
Fam198b	Up	1.03435	0.0249	chr3:79859292‐79946280	+
Kif14	Up	1.38504	0.02075	chr1:136181473‐137052008	+
Rpusd2	Up	1.38904	0.00005	chr2:119034789‐119039769	+
Slc1a2	Up	2.11536	0.00355	chr2:102658658‐102790784	+
Orai2	Up	1.01472	0.00005	chr5:136147463‐136170656	−
Ephb2	Up	1.10499	0.00005	chr4:136647538‐136835988	−
Pgap1	Up	1.28283	0.00005	chr1:54472999‐54557684	−
Xrcc2	Up	1.12742	0.0106	chr5:25582629‐26169460	−
Zfp37	Up	1.03346	0.00145	chr4:62189539‐62208446	−
Paqr8	Up	1.481	0.00005	chr1:20890621‐20938755	+
Steap2	Up	1.0385	0.00015	chr5:5664830‐5694578	−
Klrk1	Down	−1.62061	0.00905	chr6:129610322‐129623864	−
Nebl	Down	−1.49013	0.0299	chr2:17343908‐17731464	−
Sh2d7	Down	−1.65355	0.00025	chr9:54538983‐54560218	+
Cd84	Down	−1.0478	0.0017	chr1:171839696‐171890718	+
Siglech	Down	−2.62999	0.0001	chr7:55768177‐55778925	+
Sprr2a1	Down	−1.46984	0.00005	chr3:92215834‐92257298	+
Strip2	Down	−1.15512	0.0007	chr6:29917012‐29959681	+
Hpgds	Down	−1.10104	0.023	chr6:65116828‐65144908	−
Cd28	Down	−1.83097	0.00005	chr1:60716799‐60773359	+
Inpp5d	Down	−1.15829	0.0106	chr1:87620311‐87720868	+
Spib	Down	−1.15289	0.0007	chr7:44525994‐44532071	−
Ptpn7	Down	−1.49671	0.00005	chr1:135132724‐135145326	+
Cd4	Down	−1.08809	0.00055	chr6:124864691‐124888221	−
Kctd14	Down	−1.10169	0.0078	chr7:97451333‐97459553	+
Traf1	Down	−1.44914	0.0017	chr2:34941749‐34961772	−
Srgap3	Down	−1.44089	0.0004	chr6:112717970‐112947266	−
Itga4	Down	−1.25717	0.00005	chr2:79255425‐79461107	+
P2rx7	Down	−1.00178	0.00165	chr5:122643910‐122691432	+
Sypl2	Down	−1.35138	0.0007	chr3:108211471‐108226648	−
Sh2d2a	Down	−2.02063	0.00005	chr3:87846754‐87855722	+
Vwa1	Down	−1.10347	0.00005	chr4:155761177‐155776161	−
Ikzf2	Down	−2.00808	0.00005	chr1:69513931‐69685960	−
Prkcb	Down	−1.76677	0.00005	chr7:122288750‐122634402	+
Ppp1r16b	Down	−1.21456	0.00005	chr2:158665397‐158766334	+
St6galnac3	Down	−1.44486	0.009	chr3:153205404‐153725062	−

### 3.2 Validation of lncRNAs and mRNAs expression using qRT‐PCR

**Figure 4:**
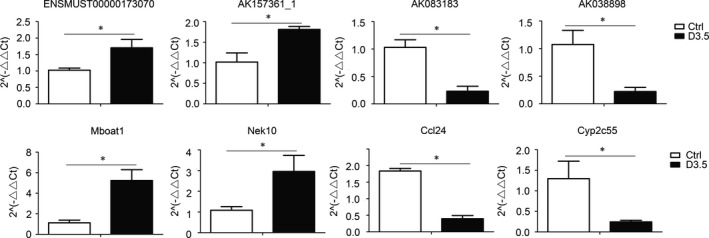
qRT‐PCR validations of two up‐regulated and two down‐regulated lncRNAs and mRNAs. ENSMUST00000173070 and AK157361 are two up‐regulated lncRNAs, AK083183 and AK038898 are two down‐regulated lncRNAs, Mboat1 and Nek10 are two up‐regulated mRNAs, Ccl24 and Cyp2c55 are two down‐regulated mRNAs. *P < 0.05

We selected two up‐regulated, two down‐regulated lncRNAs and mRNAs at 3.5 days post‐irradiation for validation purposes. Our qRT‐PCR results indicated that up‐ and downregulated lncRNAs and mRNAs were consistent with our sequencing results (Figure 4).

### 3.3 Functional analysis of miRNA target genes

**Figure 5:**
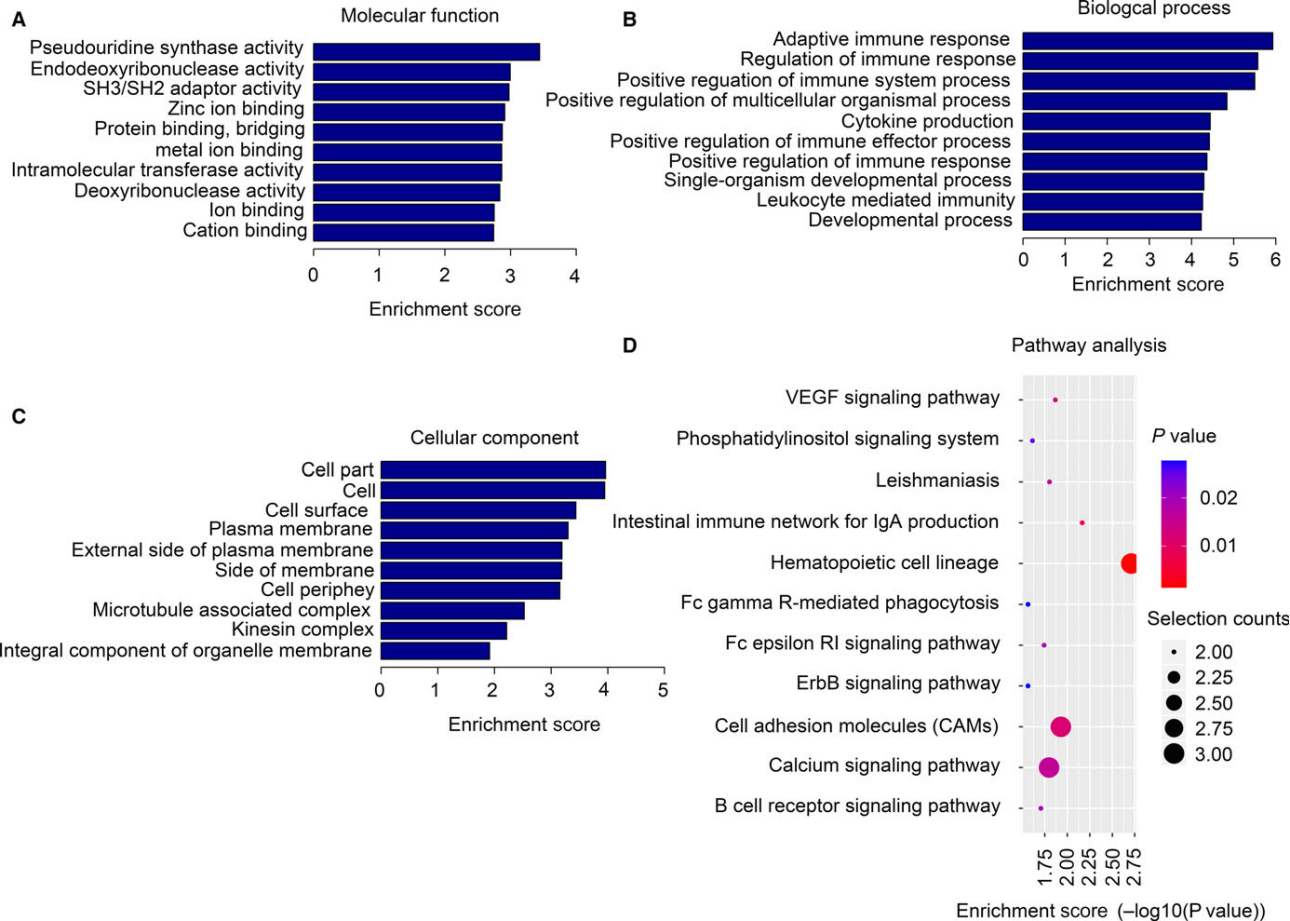
GO and KEGG signalling pathway analyses of differentially expressed lncRNAs‐targeted mRNAs. (A), Molecular function (MF), (B) Biological process (BP) and (C) Cellular component (CC) of lncRNAs‐targeted mRNAs. (D), KEGG signalling pathway analysis of lncRNAs‐targeted mRNAs

We next performed a GO analysis to better understand the functional association of target genes with the differentially expressed lncRNAs (Figure 5). Our GO analysis included three parts: MF, BP and CC. As shown in Figure 5A, we determined two main functions for the first part (MF): Pseudouridine synthase activity and endodeoxyribonuclease activity. Their function with regard to biological processes (Figure 5B) was determined to be adaptive immune response and regulation of the immune response. When classified according to their respective CC classifications (Figure 5C), the cell part and cell make up the largest proportion.

The function of the predicted target mRNAs of the identified lncRNAs was next analysed using a KEGG pathway analysis (Figure 5D). The differentially expressed lncRNAs and their target mRNAs were determined to be involved in the following biological and cellular functions: VEGF signalling pathway, phosphatidylinositol signalling system, leishmaniasis and the intestinal immune network for IgA production. With regard to the VEGF signalling pathway, two predicted mRNAs were shown to be involved: Prkcb and Sh2d2a. Prkcb is also involved in phosphatidylinositol and ErbB signalling systems. Interestingly, work using an experimental chronic colitis model induced by DSS has shown that VEGF‐C can reduce intestinal inflammation by regulating IL‐9/IL‐17 balance and improving the gut microbiota.^28^

### 3.4 Prediction of miRNA binding sites and lncRNA‐miRNA‐mRNA network analysis

**Figure 6:**
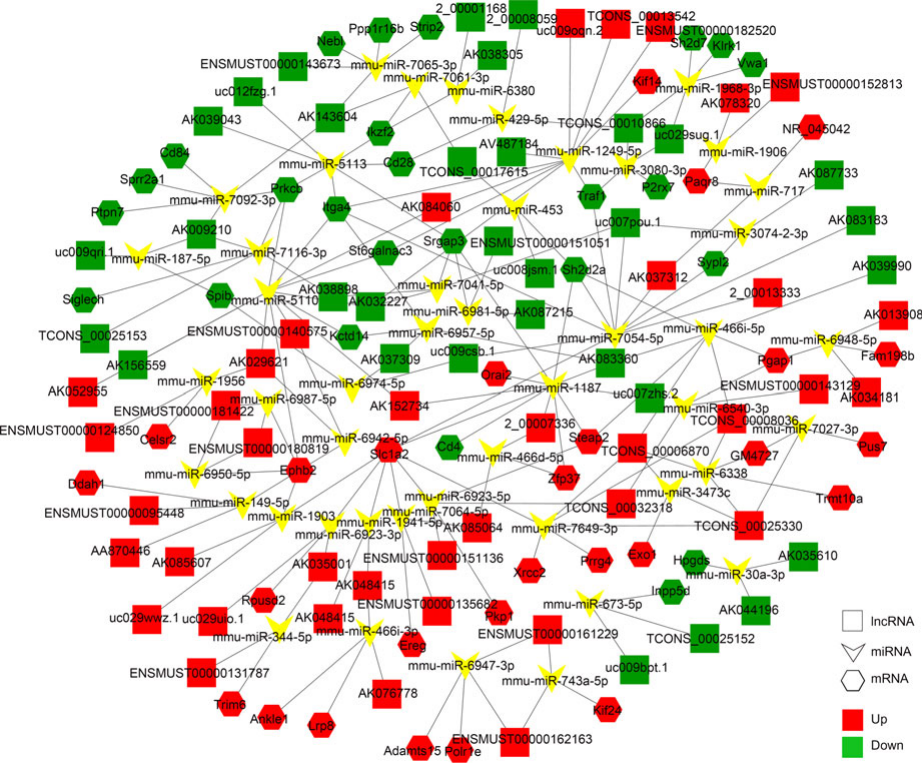
lncRNA‐miRNA‐mRNAs regulatory network analysis of lncRNAs‐targeted mRNAs. The quadrilateral represents lncRNAs, arrowheads represent miRNAs, hexagons represent mRNAs, red represents up‐regulated, and green represents down‐regulated

We selected up‐regulated lncRNAs and their 26 targeted mRNAs as well as down‐regulated lncRNAs and their 25 targeted mRNAs to construct our lncRNAs‐miRNAs‐mRNAs network (Figure 6). As indicated, the network is complex‐ one lncRNA can associate with multiple miRNAs and one miRNA can inhibit multiple mRNAs. The miRNA mmu‐miR‐5110 combined the most lncRNAs and target mRNAs. Critically, some of these targeted mRNAs have been reported to be involved in the radiation‐induced intestinal injury or repair. Ephb2 is an intestinal stem cell marker, and it has been studied extensively in colorectal cancer.^29^ It has been proposed that EXO1 acts in the excision step during mismatch‐repair. After mismatch recognition in prokaryotes and eukaryotes,^30^ EXO1 is also involved in the 5′ to 3′ end resection at DSB (double‐strand breaks) ends to initiate HR in both yeast and mammalian systems.^31^ Moreover, Kobayashi et al^32^ found that Pgap1 was highly expressed by the follicle‐associated epithelium; given this, it may be associated with intestinal mucosal immunity. A better understanding of lncRNA function, as well as their potential intestinal target genes will need further study.

## 4 DISCUSSION

Here, we provide a basic study of lncRNAs function during the radiation‐induced intestinal injury. Using a sequencing approach, we obtained 29 007 lncRNAs from the control and irradiated groups. Of these 29 007 lncRNAs, there were 2510 lncRNAs detected only at 3.5 days post‐irradiation, while 23 807 were detected in both groups. After comparing the irradiated and control groups, we found 91 lncRNAs were significantly up‐regulated and 58 were significantly down‐regulated after irradiation.

We also obtained 17 142 mRNAs, of which 346 were only detected 3.5 days post‐irradiation and 16 514 were detected in both the groups. Comparing the two groups revealed that 752 mRNAs were significantly up‐regulated and 400 were significantly down‐regulated in the irradiated group when compared with controls.

We selected the 91 significantly up‐ and 58 down‐regulated lncRNAs, predicted their combined miRNAs and miRNA‐targeted mRNAs, and compared these with our mRNAs sequencing results, selected both lncRNAs and target mRNAs up‐regulated and down‐regulated. These lncRNAs may act as an endogenous “sponge” that regulates the target gene of miRNAs through competition with miRNAs. By comparing our predicted results with those obtained through sequencing, we found up‐regulated lncRNAs‐targeted 26 mRNAs and down‐regulated lncRNAs‐targeted 25 mRNAs. Due to consistent regulation with mRNAs, lncRNAs may function as a miRNAs sponge.

Using these data, we next performed a GO analysis and found that their biological function was relegated to an adaptive immune response and regulation of the immune response. As is well known, the intestinal mucosa harbours one of the largest, most complex immune systems in the body. Even under normal conditions, the intestinal mucosa exhibits a state of “physiological inflammation” in response to dietary and bacterial antigens that are present in the intestinal lumen.^33^ When exposed to radiation, immune cells are activated and inflammatory cell recruitment occurs. The result is that activated immune cells attack and destroy neighbouring cells. Given this biological function, it is likely that some genes may regulate the radiation‐induced intestinal injury through regulation of the immune response.

Our KEGG signalling pathway analysis showed that the differentially expressed lncRNAs‐targeted mRNAs were mainly involved in VEGF signalling. The VEGF signalling pathway is a well‐studied pathway that is involved in angiogenesis; researchers have also found that this pathway plays an important role in intestinal immune and inflammation. Chronic, intestinal inflammation is associated with pathological angiogenesis that further amplifies the inflammatory response. For instance, Ardelean^34^ showed that a VEGF monoclonal antibody could ameliorate chronic colitis induced by DSS in *Endoglin* heterozygous mice. Schlieve et al^35^ found that manipulating VEGF bioavailability led to profound effects on not only intestinal vasculature, but the epithelial stem and progenitor cells in the intestinal crypt. When taken together, these findings indicate a potential role for VEGF in the radiation‐induced intestinal injury.

The lncRNAs‐miRNAs‐mRNAs network is very complex‐ one lncRNA had multiple miRNA binding sites and could combine with multiple lncRNAs while one miRNA also combined via multiple lncRNAs. lncRNAs target different miRNAs and play different roles. Importantly, even lncRNAs from the same gene may play different roles. For example, the MBNL3^36^ splicing factor induces lncRNA‐PXN‐AS1 exon 4 inclusion. The transcript‐lacking exon 4 binds to the PXN mRNA coding sequences, causing dissociation of translation elongation factors from PXN mRNA. This leads to inhibition of PXN mRNA translation. In contrast, the transcript containing exon 4 preferentially binds to the 3′ untranslated region of the PXN mRNA, and protecting PXN mRNA from microRNA‐24‐AGO2 complex‐induced degradation. This leads to increased PXN expression. We have performed sequencing on jejuna samples post‐irradiation and analysed the resulting lncRNAs. However, we only predicted the sponge function of lncRNAs with their target mRNAs and all will need to be verified. In addition to its miRNA sponge function, lncRNAs can also form complementary double strands with protein‐encoding transcripts, interfere with mRNA splicing and/or bind and modulate protein activity. Further work will be needed to better elucidate lncRNA function.

More than 70% of cancer patients undergo radiotherapy as intestinal mucosa undergoes rapid and constant renewal throughout the life of the organism. It is hard to protect and the intestinal epithelium is particularly sensitive to ionising radiation. Radiation‐induced intestinal toxicity results in diarrhoea, dehydration and even death. Despite this, there is currently no accepted approach to prevent or treat the radiation‐induced intestinal injury. Although our laboratory has found that mesenchymal stem cell^37^, ^38^ and 6‐shogaol^39^ can improve animal survival and intestinal function following irradiation injury, we still need to understand more about the effects of radiation in order to solve the problem of radiation‐induced intestinal damage from the effect itself. Therefore, our previous work has analysed the regulatory network of circRNAs^40^ in radiation‐induced intestinal injury, as well as some important responses of mRNAs.^41^ In this article, we focus on the role of lncRNAs. Researchers have found lncRNAs play an important role in ontogenesis and disease occurrence. As mentioned previously, many lncRNAs are involved in maintaining and regulating the intestinal epithelial barrier and DNA damage induced by ionising radiation. However, there has been little work carried out regarding the role of lncRNAs in the radiation‐induced intestinal injury or repair. We hope that the identification of lncRNAs that regulate the radiation‐induced intestinal injury and repair will help make a contribution to better approaches for the clinical treatment of the radiation‐induced intestinal injury.

## CONFLICT OF INTEREST

We declare that we have no financial and personal relationships with other people or organizations that can inappropriately influence our work. There is no professional or other personal interest of any nature or kind in any product, service and/or company that could be construed as influencing the position presented in, or the review of, the manuscript entitled.
